# Efficient Promotion of Autophagy and Angiogenesis Using Mesenchymal Stem Cell Therapy Enhanced by the Low-Energy Shock Waves in the Treatment of Erectile Dysfunction

**DOI:** 10.1155/2018/1302672

**Published:** 2018-08-29

**Authors:** Guan Qun Zhu, Seung Hwan Jeon, Woong Jin Bae, Sae Woong Choi, Hyun Cheol Jeong, Kang Sup Kim, Su Jin Kim, Hyuk Jin Cho, U. Syn Ha, Sung Hoo Hong, Ji Youl Lee, Eun Bi Kwon, Sae Woong Kim

**Affiliations:** ^1^Department of Urology, College of Medicine, The Catholic University of Korea, Seoul, Republic of Korea; ^2^Catholic Integrative Medicine Research Institute, College of Medicine, The Catholic University of Korea, Seoul, Republic of Korea; ^3^Department of Urology, Wonju College of Medicine, Yonsei University, Wonju, Republic of Korea; ^4^KEMIMEDI, Seoul, Republic of Korea

## Abstract

**Background:**

Mesenchymal stem cell therapy (MSCT) and defocused low-energy shock wave therapy (ESWT) has been shown to ameliorate erectile dysfunction (ED). However, the interactions and effects of action between MSCT and ESWT remain poorly understood. In this study, we investigated the mechanisms of combination therapy with MSCT and ESWT in a rat model of diabetic ED.

**Materials and Methods:**

Eight-week-old male Sprague-Dawley rats were randomly divided into 2 parts. Diabetic rats induced by streptozotocin (65 mg/kg) were randomly divided into 4 groups: (1) DM control group, (2) DM + ESWT group, (3) DM + MSCT group, and (4) DM + ESWT + MSCT group. The sham group was a normal control group (without streptozotocin). MSCT and (or) ESWT were, respectively, administered to each group according to the proposal for 8 weeks. Immediately after recording of intracavernous pressure (ICP), the penis was then harvested for histologic analysis, ELISA, and Western blotting.

**Results:**

The ratio of ICP/MAP was significantly higher in the DM + ESWT + MSCT group than in ESWT or MSCT treated group (*P* < 0.05). Also, the treatment stimulated angiogenesis and vasodilatation in the corpus cavernosum (*P* < 0.05). ESWT increased the quantity of MSCs in the corpus cavernosum and also induced MSCs to express more VEGF in vitro and vivo (*P* < 0.05) which activated the PI3K/AKT/mTOR and NO/cGMP signaling pathways in the corpus cavernosum. The combination approach stimulated autophagy and decreased apoptosis in the corpus cavernosum. NGF and BDNF expressions were higher in the DM + ESWT + MSCT group than in the DM control group (*P* < 0.01). Furthermore, the treatment promoted the MSC recruitment by inducing penile tissues to express more PECAM and SDF-1.

**Conclusions:**

Combination of LI-ESWT and MSCT can get a better result than a single treatment by expressing more VEGF which can take part in autophagy by triggering the PI3K/AKT/mTOR signaling pathway. This cooperative therapy would provide a new research direction in ED treatment for the future.

## 1. Introduction

Diabetic patients suffer from a higher incidence of erectile dysfunction (ED), which is less responsive to drugs than nondiabetic individuals, and this diabetes mellitus (DM) erectile dysfunction (DMED) seriously influences the quality of life of diabetic patients [1–3]. Now, phosphodiesterase 5 inhibitor (PDE5I) represents the first-line treatment for ED, but there are still poor therapeutic effects for some DMED patients [4–6]. So it is extremely urgent to find a new and therapeutic approach to treat the drug-useless DMED patients.

As a relatively novel approach in regeneration medicine, defocused low-energy shock wave therapy (DL-ESWT) has shown great potential and promising evidences, especially for the treatment of various disorders such as tissue trauma and defects [7–11]. At present, ESWT has been applied to clinical therapy for ED, and many studies have shown that ESWT can achieve satisfactory therapeutic effects [12–14]. The main mechanism of ESWT is considered that can stimulate angiogenesis and restore blood flow to the disorder area, through promoting regeneration and repair [15]. Hayashi et al. [7] reported that the expressions of various proangiogenic factors, such as vascular endothelial growth factor (VEGF) and endothelial nitric oxide synthase (eNOS), could be upregulated by DL-ESWT in diabetic mice. Another reason for ED therapy using DL-ESWT is that it can enhance the recruitment of endothelial progenitor cells by upregulating stromal cell-derived factor-1 (SDF-1) in wound tissues [16]. Li et al. [17] found that DL-ESWT could promote the recruitment of endogenous progenitor cells and activation of Schwann cells, coinciding with angiogenesis, tissue, and nerve generation in a rat model of pelvic neurovascular injuries.

Now, some researchers [18–20] suggest that as a new approach, mesenchymal stem cell (MSC) therapy can be a good remedial method for ED. Gokce et al. [21] consider that the reason and mechanism of ED stem cell therapy are that stem cells can stimulate endothelial cell proliferation and inhibit endothelial cell apoptosis by a paracrine mechanism. But MSCs' survival rate is still a problem through intracavernous injection. Liu et al. [22] consider that in the DMED rat model hyperglycemia can induce cell apoptosis, but oxidative stress also can increase MSC autophagy by cell self-defense, and augmentation of autophagy can reduce apoptosis, prolong MSC survival, and improve MSC-based therapeutic efficacy for DMED. Also, some studies [23, 24] prove that not all MSCs, through intracavernous injection, will be apoptotic, dead immediately, or flowing away by migrating into the circulatory system. So we believe that mesenchymal stem cell therapy (MSCT) will be a potential and promising ED treatment.

Since both ESWT and MSCT can be an approach for ED treatment by paracrine growth factors to promote cellular proliferation, we have reasons to believe that combination of both for ED treatment will get a better result. In this report, we proved that combination of LI-EWST and MSCT might be a better way to improve ED by promoting angiogenesis, regeneration, and autophagy. Our study also proved that ESWT can drive homing of MSCs. Also, this cooperative therapy combining stem cells with machine provides a theoretic and experimental basis for clinic treatment.

## 2. Materials and Methods

### 2.1. Cell Cultures

MSCs come from the surplus stock of frozen cells at a cell bank (SL BIGEN, Seongnam, Korea). Cells were cultured in a special medium (mesenchymal stem cell proliferation media; SL BIGEN) and maintained at 37°C with 5% CO_2_ in a humidified atmosphere. Two days later, nonadherent cells were removed and fresh culture medium was added. The culture medium was changed every 2 days. Cells were passaged when they reached approximately 90% confluence. The ESWT on cultured MSCs was performed by an extracorporeal shock wave machine (Urontech, Hwaseong, Korea). The shock wave probe was kept in contact with the culture flask containing adherent MSCs by means of a water-filled cushion covered with common ultrasound gel. The cells were subjected to 200 impulses of ESWT at an energy flux density of 0.09 mJ/mm^2^ with a frequency of 120/min as described previously [25]. ESWT was performed after cell attachment. Every generation only accepted ESWT one time.

### 2.2. Effect of ESWT on Activating MSCs In Vitro

We quantified the VEGF in the cell culture supernatants by species-specific VEGF immunoassay ELISA kits (R&D Systems Europe, Abingdon, United Kingdom) according to the manufacturer's instructions. After ESWT, MSCs (each well seeded the same quantity of MSCs and from the first passage to the fifth passage of MSCs was seeded in a discrete 6 cm culture dish) were cultured for 12 h in a constant-temperature incubator, and then 1 mL of medium was collected and stored at −80°C until used. Absorbance was read at a wavelength of 450 nm in a microplate reader (Synergy H1 M, Biotek, USA). At least 3 separate dishes of cells were assayed for each clone.

### 2.3. Experimental Animal and Study Design

Eight-week-old male Sprague-Dawley rats weighing 270–300 g (Orient Bio Co., Seongnam, Korea) were used in this study. All animal experiments in this study were approved by the Institutional Animal Care and Use Committee of the Catholic University of Korea. DM was induced by an intraperitoneal injection of streptozotocin (STZ, Sigma-Aldrich chemical company, USA) at a dose of 65 mg/kg body weight (prepared STZ in 50 mM citrated buffer pH 4.5) as described previously [26], and the sham rats were injected in an equal vehicle (0.1 mol/L citrate-phosphate buffer, pH 4.5). After 72 h, DM was confirmed by blood glucose levels above 300 mg/dL to determine by the Accu-Chek Active blood glucose monitor (Hoffmann-La Roche Ltd., Basel, Switzerland), and blood samples were obtained from tail veins (the percentage of diabetic animals after STZ injection was about 70%). Unhealthy rats showing ruffled hair, poor appearance, vocalization, and lack of appetite were not included in our experiment. After 12 weeks, evaluation of erectile function was performed for these rats and rats with normal erectile function were excluded. In brief, streptozotocin-induced rats were put into a rearing cage with an adult female rat. If seminal stain was not found in the cage in 1 week, the experimental rat was included in the next process. If seminal stain was found, the rat was excluded. The included rats were randomly divided into 4 groups (*N* = 10 per group): (1) DM control group, (2) DM + ESWT group, (3) DM + MSCT group, and (4) DM + ESWT + MSCT group, and the sham group (*N* = 10, without STZ) was the normal control group.

### 2.4. MSC Injection and ESWT

All experimental rats (including DM control, DM + ESWT, DM + MSCT, and DM + ESWT + MSCT groups) underwent bilateral intracavernous injection. Briefly, animals in experimental groups were anesthetized by inhalational 2.0% isoflurane, and then each rat was placed in the back position with its lower abdomen shaved and the penis was drawn out of the prepuce. Before MSC injection, we labeled the MSCs with a fluorescent dye (CellTracker™ CM-DiI; Molecular Probes, Eugene, OR) to track the location of MSCs according to the manufacturer's protocol. Rats in DM + ESWT and DM + ESWT + MSCT groups received bilateral intracavernous injection of 200 *μ*L PBS solution containing 1 × 10^6^ MSCs (P3) as described previously [23, 27], and the DM control and DM + ESWT groups received bilateral intracavernous injection of just 200 *μ*L PBS. MSCT was repeated 1 time per week, for a total duration of 4 weeks.

After MSC injection, rats in the DM + ESWT and DM + ESWT + MSCT groups were treated with shock waves. Under anesthesia, the penis was drawn out of the prepuce in a supine position. Ultrasonic gel was applied to the penis, and then the shock wave applicator (Urontech, Hwaseong, Korea) was placed on the penis. Each penis was subjected to 300 impulses of ESWT at an energy flux density of 0.1 mJ/mm^2^ with a frequency of 120/min which is based on other studies [17, 28]. ESWT was repeated 3 times per week with one day's break, for a total duration of 4 weeks.

### 2.5. Erectile Function Evaluation

Under anesthesia, the carotid artery and cavernous nerve were exposed via midline laparotomy. The corpus cavernosum of the penis was cannulated with a 25-gauge butterfly needle filled with heparin (250 U/mL) via insertion at the crura. The cannula was connected to a pressure transducer (Grass model S48 K; Astro-Med Inc., West Warwick, RI) for continuous assessment and recording of intracavernous pressure (ICP). BD Intramedic PE-50 tubing (BD, Franklin Lakes, NJ) was inserted into the carotid artery for the measurement of MAP. Both ICP and mean arterial blood pressure (MAP) were recorded continuously as previously described [23, 29, 30]. The stimulus parameters were 1.5 mA, 20 Hz, pulse width 0.2 ms, and duration 50 s. The maximal ICP during nerve electrostimulation was calculated from an isometric force transducer and recorded on a computer with a PowerLab commercial data acquisition system (AD Instruments, Dunedin, New Zealand). The ratio of ICP to MAP was used to evaluate the level of erectile function. The penis was then harvested for histologic analysis, ELISA, and Western blotting.

### 2.6. Histology and Immunofluorescence Staining

Animals were sacrificed by intraperitoneal injection with sodium pentobarbital (50 mg/kg), followed by bilateral thoracotomy. Penile midshaft tissues were freshly harvested and fixed [23], and immunofluorescence staining was performed as previously described [31]. The corpus cavernosum paraffin sections were immunostained with VEGF (diluted 1 : 200 Santa Cruz Biotechnologies, Santa Cruz, US), neuronal nitric oxide synthase (nNOS, diluted 1 : 200 Santa Cruz Biotechnologies, Santa Cruz, CA), brain-derived neurotrophic factor (BDNF, diluted 1 : 200; Abcam, Cambridge, UK), nerve growth factor (NGF, diluted 1 : 200; Abcam, Cambridge, UK), stromal cell-derived factor-1 (SDF-1 diluted 1 : 200; Abcam, Cambridge, UK), and neuron-specific *β*-III tubulin (diluted 1 : 200; Abcam, Cambridge, UK) and mounted with 4,6-diamidino-2-phenylindole (DAPI; Vector Laboratories Inc., Burlingame, CA) to stain the nuclei. The collagen and vascular smooth muscle was evaluated after tissue staining with the Masson trichrome technique using the method previously described [32]. Digital images were obtained using a Zeiss LSM 510 Meta confocal microscope (Zeiss, Oberkochen, Germany), and the mean intensity was calculated using ZEN 2009 (Zeiss).

### 2.7. Analysis of Apoptosis and Autophagy In Vivo

We assessed the apoptosis in vivo using Western blotting to test the poly-ADP-ribose polymerase (PARP) as previously described [33]. We also analyzed the autophagy in penile tissue by Western blotting to detect light chain 3 (LC3) [34]. The antibodies of PARP and LC3 were listed in Western blot analysis.

### 2.8. Effect of Combination between ESWT and MSCT In Vivo

60 mg of penile tissue from both normal control and experimental animals was immersed with 350 mL of 0.1 M HCL, and silica beads were added (BioSec Enviro Inc., Guelph, Canada). The treated sample was homogenized in 2 mL lysate buffer (Precellys 24; Bertin Technologies, Montigny-le-Bretonneux, France) and centrifuged at 14,000 ×g for 10 minutes at 4°C, and then its supernatant was extracted. The protein concentration in each sample was determined by bicinchoninic acid (BCA) assay according to the manufacturer's instructions. The cGMP (cyclic guanosine monophosphate) direct immunoassay kit (K372, BioVision, San Francisco, US) was used for detection of cavernous cGMP levels according to the manufacturer's protocol.

### 2.9. Western Blot Analysis

Corpus cavernosum tissue was homogenized using ice-cold RIPA buffer (Cell Signaling Technology, Danvers, US) containing ethylenediaminetetraacetic acid-free protease inhibitor cocktail and phosphatase inhibitor cocktail (Roche Diagnostics GmbH). The homogenized sample was then centrifuged at 12,000 ×g for 10 minutes at 4°C and its supernatant extracted. This supernatant was electrophoresed on NuPAGE 4%–12% bis-Tris gel (Invitrogen, Carlsbad, CA) and then transferred onto a nitrocellulose membrane. After the transfer, the membrane was blocked with 5% skim milk at room temperature for 1 hour and then incubated with the primary antibodies including endothelial nitric oxide synthase (eNOS diluted 1 : 500 Santa Cruz Biotechnologies, Santa Cruz, CA), phosphorylated endothelial nitric oxide synthase (p-eNOS diluted 1 : 500 Santa Cruz Biotechnologies, Santa Cruz, CA), AKT (diluted 1 : 500; Cell Signaling Technology, Danvers, US), p-AKT (diluted 1 : 500; Cell Signaling Technology, Danvers, US), PARP (diluted 1 : 500; Abcam, Cambridge, UK), LC3 (diluted 1 : 500; Abcam, Cambridge, UK), platelet endothelial cell adhesion molecule (PECAM, diluted 1 : 500; Abcam, Cambridge, UK), and *β*-actin (diluted 1 : 1000; Abcam, Cambridge, UK). After this, the membrane was incubated with a secondary antibody conjugated to horseradish peroxidase for 1 hour at room temperature. The enhanced chemiluminescence method (Amersham, Arlington Heights, IL) was used for protein detection. The resulting images were analyzed using ImageJ (National Institutes of Health, Bethesda, MD, US) to determine the integrated density for each protein band.

### 2.10. Image and Statistical Analysis

The images were quantified by ImageJ and Image-Pro Plus (Media Cybernetics, Silver Spring, MD, USA). The results were analyzed using SPSS 20.0 software (SPSS Inc., Chicago, IL, USA). The measurement data are presented as mean ± standard deviation (SD), and the multigroup comparisons were made with (ANOVA) followed by the Tukey-Kramer test for post hoc comparisons. Data expressed as proportions were assessed with a chi square test. Values of *P* < 0.05 were considered to indicate a statistically significant difference.

## 3. Results

### 3.1. ESWT + MSCT Can Improve ED Significantly

**Figure 1:**
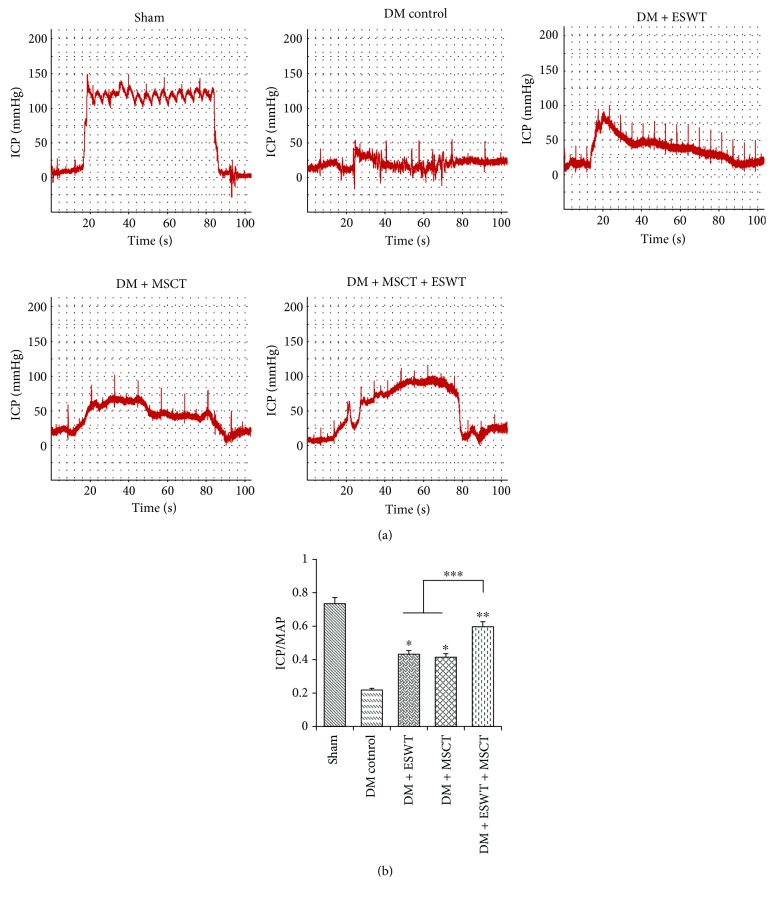
Erectile function of each group. (a) Representative images of intracavernous pressure (ICP) in response to electrical stimulation of the cavernosal nerve. (b) Results of ratio ICP to mean MAP of each group. Each bar shows the mean values (standard deviation). ^∗^ *p* < 0.05 compared with DMED rats. ^∗∗^ *p* < 0.01 compared with DMED rats. ^∗∗∗^ *p* < 0.05 compared with DM + ESWT and DM + MSCT groups.

ICP results are shown in Figure 1. As we can see from Figure 1(a), the ICP result of the DM + ESWT + MSCT group is apparently higher than the result of the DM control group. In quantitative results (Figure 1(b)), the sham group is 0.73 ± 0.08. The experimental groups including DM control, DM + ESWT, DM + MSCT, and DM + ESWT + MSCT groups are, in order, 0.21 ± 0.08, 0.43 ± 0.07, 0.41 ± 0.11, and 0.59 ± 0.09. Both ESWT and MSCT can improve ED; their quantitative results are higher than in the DM control group (*P* < 0.05). The ratio of ICP/MAP was significantly higher in the DM + ESWT + MSCT group than in the DM + ESWT and DM + MSCT groups (*P* < 0.05). The quantitative result of the DM + ESWT + MSCT group is very close to the result of the sham group. So we can hold the opinion that ED treatment is more effective by DM + ESWT + MSCT than by a single way (ESWT or MSCT).

### 3.2. ESWT + MSCT Stimulates Angiogenesis and Vasodilatation in DMED Rats' Corpus Cavernosum

**Figure 2:**
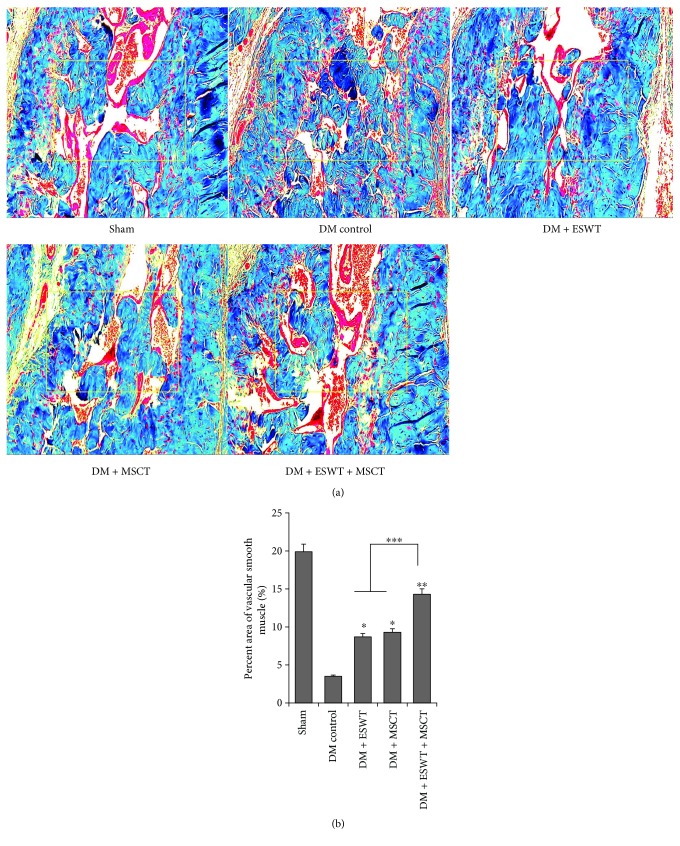
Masson staining of corpus cavernosum (a) Representative images of Masson trichrome stain for each group. Red is vascular smooth muscle, and blue is collagen. Original magnification: ×200. (b) Percentage area of endothelial cell smooth muscle cells for each group. Each bar shows the mean values (standard deviation). ^∗^ *P* < 0.05 compared with the DM control group. ^∗∗^ *P* < 0.01 compared with the DM control group. ^∗∗∗^ *P* < 0.05 compared with the DM + ESWT and DM + MSCT groups.

The result of Masson staining (Figure 2(a)) shows that the vascular smooth muscle of the DM + ESWT + MSCT group is obviously more, in comparison with vascular smooth muscle of the DM group. These results show that both EWST and MSCT can stimulate angiogenesis, compared with the DE group (*P* < 0.05). The result was significantly higher in the DM + ESWT + MSCT group than in the DM + ESWT or DM + MSCT groups (*P* < 0.05), which proved that DM + ESWT + MSCT could stimulate angiogenesis and vasodilatation, significantly. It is also emphasized that angiogenesis and vasodilatation (Figure 2(a)) in the DM + ESWT + MSCT group is approximated to the sham group. One of the primary reasons for DM is vascular damage caused by oxidative stress response and hyperglycemia [35, 36]. Insufficient blood supply results from vascular damage leading to ED. So increasing the angiogenesis and recovering vascular function are key to restoring erectile function. Now, PDE5I is the first-line treatment for ED, but there are still poor therapeutic effects for some DMED patients especially with damaged vascular endothelium [4–6]. As we can see from our results (Figure 2), ESWT + MSCT can obviously increase the blood supply in the corpus cavernosum. So we believe that after ESWT + MSCT, the erectile function of DMED patients will be improved significantly.

### 3.3. ESWT Increases the Quantity of MSCs in the Corpus Cavernosum

**Figure 3:**
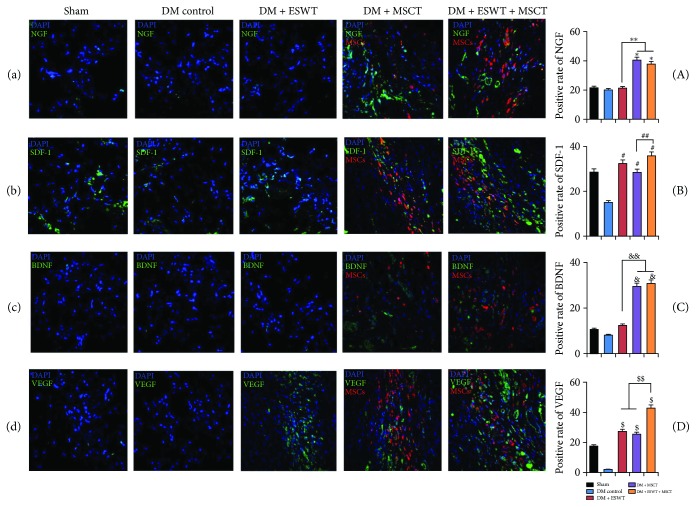
ESWT + MSCT promoted proliferation and migration in vivo. (a) Representative images of NGF stain for each group. Original magnification: ×200. (B) Positive rate of NGF for each group. Each bar shows the mean values (standard deviation). ^∗^ *P* < 0.01 compared with the DM control group, ^∗∗^ *P* < 0.01. (b) Representative images of SDF-1 stain for each group. Original magnification: ×200. (B) Positive rate of SDF-1 for each group. Each bar shows the mean values (standard deviation). ^#^ *P* < 0.01 compared with the DM control group, ^##^ *P* < 0.05. (b) Representative images of BDNF stain for each group. Original magnification: ×200. (B) Positive rate of BDNF for each group. Each bar shows the mean values (standard deviation). ^&^ *P* < 0.01 compared with the DM control group, ^&&^ *P* < 0.01. (b) Representative images of VEGF stain for each group. Original magnification: ×200. (b) Positive rate of VEGF for each group. Each bar shows the mean values (standard deviation). ^$^ *P* < 0.01 compared with the DM control group, ^$$^ *P* < 0.05.

**Figure 4:**
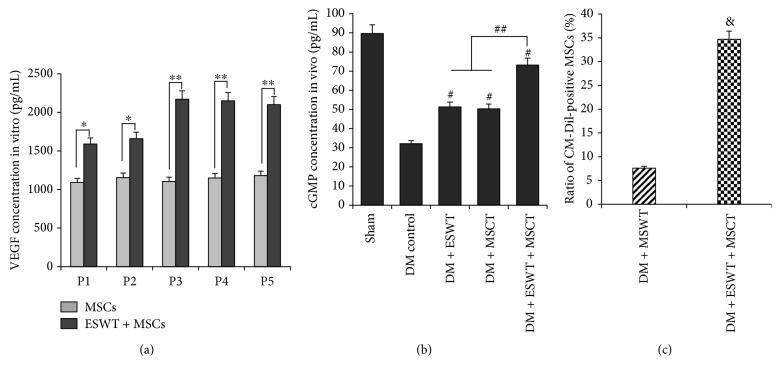
(a) VEGF concentration in vitro from P1 to P5 after ESWT or not, by ELISA. ^∗^ *P* < 0.05, with untreated MSCs; treated MSCs secreted higher levels of VEGF. ^∗∗^ *P* < 0.01, with untreated MSCs; treated MSCs secreted higher levels of VEGF. (b) cGMP concentration of each group in vivo, by ELISA. ^#^ *P* < 0.05, with untreated MSCs; treated MSCs secreted higher levels of cGMP. ^#^ *P* < 0.05 compared with the DM control group. ^##^ *P* < 0.05 compared with the DM + ESWT and DM + MSCT groups. (c) Ratio of CM-Dil-positive MSCs in the corpus cavernosum. ^&^ *P* < 0.01; in the DM + ESWT + MSCT group, the quantity of MSCs is higher than in the DM + MSCT group.

After MSC intracavernous injection, we made a MSCT successively. In our results (Figure 3), we found that ESWT can increase the quantity of MSCs in the corpus cavernosum obviously. So we made a quantitative analysis of staining results. As we can see from the quantitative results (Figure 4(c)), we found after MSC intracavernous injection that the quantity of MSCs with MSCT was higher than the quantity of MSCs without EWST (*P* < 0.05). This result tells us that ESWT can increase MSCs.

### 3.4. ESWT Can Induce MSCs to Express More VEGF In Vitro and In Vivo

We found after ESWT that MSCs could express more VEGF (Figure 4(a)) than without ESWT in vitro (*P* < 0.05). In order to research the effect of ESWT on the MSCs in vivo, we injected MSCs into the corpus cavernosum of DMED rats and then carried out ESWT. From the results (Figures 3(a) and 3(D)), we found that VEGF expression in the DN + DMED + ESWT group was higher than others (*P* < 0.05). So we consider that after ESWT, the MSCs can express more VEGF in vivo. From Figures 3(D) and 3(d), we found that penile tissues of DMED rats scarcely expressed VEGF. This phenomenon leads to endothelial proliferation slowly and angiogenesis barely. But in the DM + ESWT or DM + MSCT groups, there was still some VEGF in the corpus cavernosum. This is one reason why ESWT and MSCT can improve DMED.

### 3.5. VEGF Activates the PI3K/AKT/mTOR and NO/cGMP Signaling Pathway in the Corpus Cavernosum

**Figure 5:**
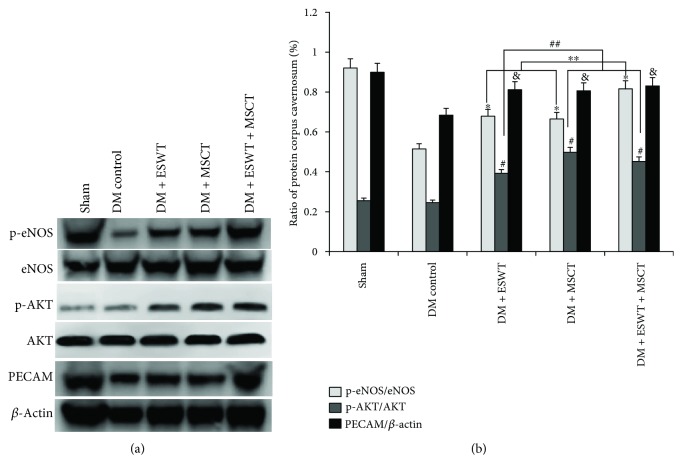
ESWT increases proteins of proliferation, autophagy, and migration. (a) All groups were compared for p-eNOS, eNOS, p-AKT, AKT, and PECAM in the corpus cavernosum using Western blotting. (b) Quantity analysis of Western blotting, including p-eNOS/eNOS, p-AKT/AKT, and PECAM/*β*-actin. ^∗^ *P* < 0.05 compared with values from the DM control group. ^∗∗^ *P* < 0.05 compared with the DM + ESWT and DM + MSCT groups. ^#^ *P* < 0.05 compared with the DM control group. ^##^ *P* < 0.05 compared with the DM + MSCT and DM + ESWT + MSCT groups. ^&^ *P* < 0.05 compared with the DM control group.

**Figure 6:**
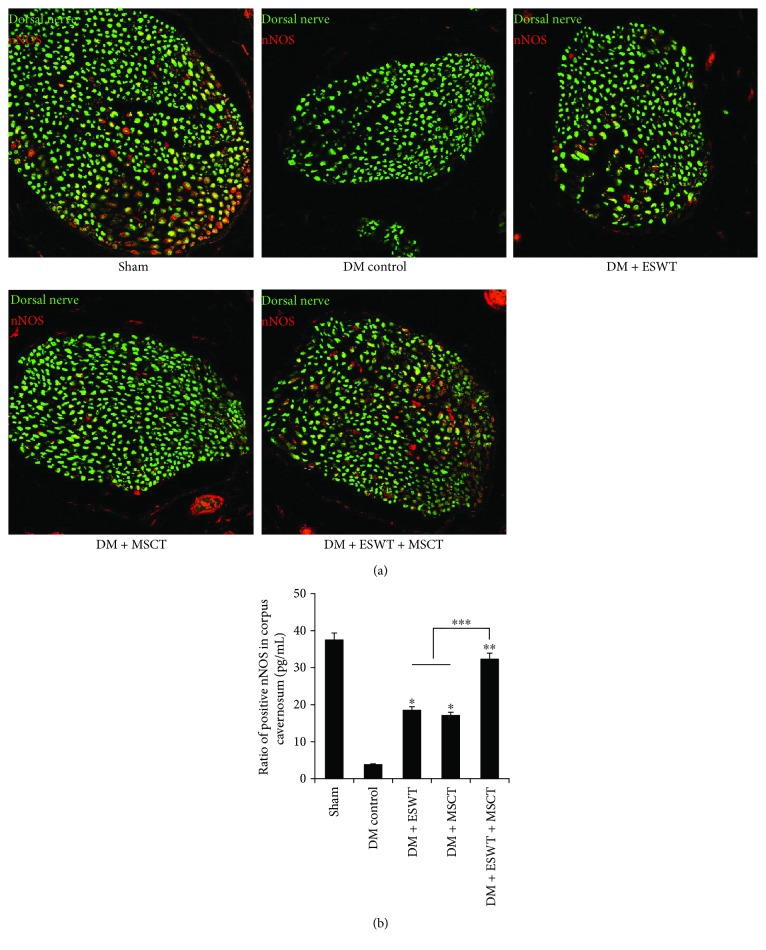
ESWT + MSCT induce nNOS expression in the corpus cavernosum. (a) Representative images of nNOS staining in the corpus cavernosum. (b) Ratio of positive nNOS of each group. Each bar shows the mean values (standard deviation). Original magnification: ×400. ^∗^ *P* < 0.05 compared with the DM control group. ^∗∗^ *P* < 0.01 compared with the DM control group. ^∗∗∗^ *P* < 0.05 compared with the DM + ESWT and DM + MSCT groups.

Some researchers [37, 38] believe that VEGF can activate the PI3K/AKT/mTOR pathway, so we detected the expression quantity of AKT and p-AKT. The results (Figures 5(a) and 5(b)) show that with ESWT + MSCT, the expression quantity of p-AKT/AKT is apparently higher than others (*P* < 0.05). Combining with the result (Figure 3(D)), we consider after ESWT + MSCT that plentiful VEGF triggers the PI3K/AKT/mTOR pathway in the DMED corpus cavernosum. Some researchers [39] consider that NO mediates vasodilatation by increasing soluble guanylate cyclase (sGC) in the smooth muscle of vessels. According to the NO/cGMP pathway [40], we detected the quantity of eNOS/p-eNOS, nNOS, and cGMP expression. The results (Figures 4(b), 5, and 6) indicate that with ESWT + MSCT, the quantity of p-eNOS/eNOS and cGMP is apparently higher than others (*P* < 0.05). Combining with the result (Figure 3(D)), we believe that, after ESWT + MSCT, much of VEGF activates the NO/cGMP pathway. As in the above, we believe that ESWT + MSCT can increase the expression of VEGF, and plenty of VEGF can trigger the PI3K/AKT/mTOR and NO/cGMP pathways.

### 3.6. ESWT + MSCT Can Stimulate Autophagy and Decrease Apoptosis in the Corpus Cavernosum

**Figure 7:**
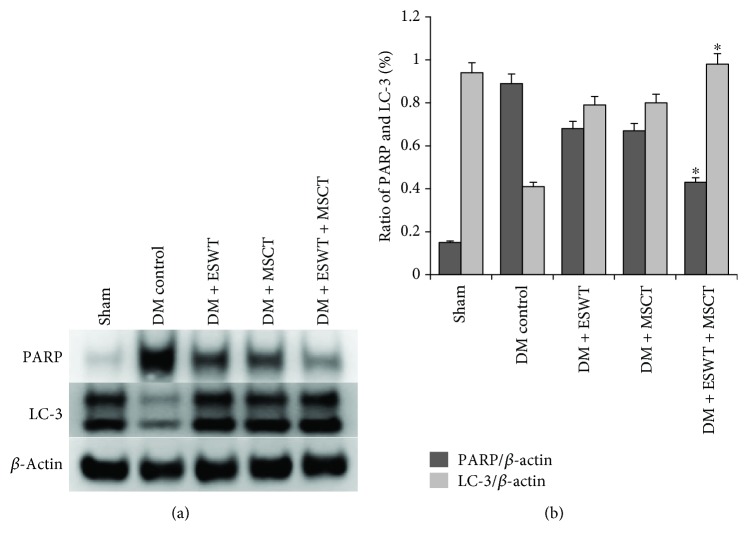
ESWT can activate autophagy and decrease apoptosis in vivo. (a) All groups were compared for PARP and LC-3 in vivo by Western blotting. (b) Quantity analysis of Western blotting, including PARP/*β*-actin and LC-3/*β*-actin. ^∗^ *P* < 0.01 compared with values from the DM control group.

Some researchers [20, 41, 42] believe that activating the PI3K/AKT/mTOR pathway can induce autophagy by reducing mechanistic target of rapamycin complex (mTORC) 1 kinase activity and Rheb levels. As a type of self-defense, autophagy can offset losses caused by apoptosis [22]. We found (Figure 4(c)) that after ESWT, the quantity of MSCs increased significantly (*P* < 0.05). So we made a hypothesis that ESWT induced MSCs to express more VEGF, and then VEGF triggered the PI3K/AKT/mTOR pathway for increasing autophagy. The results of autophagy and apoptosis assay (Figure 7) show that ESWT can activate autophagy of the penile tissues and decrease apoptosis effectively. Also, we found that NGF (Figures 3(a) and 3(A)) and BDNF (Figures 3(c) and 3(C)) expression was higher in the DM + ESWT + MSCT group than in the DM group (*P* < 0.01), which has been considered to defend apoptosis [43, 44]. These results proved our hypothesis that ESWT could stimulate autophagy and decrease apoptosis in the corpus cavernosum.

### 3.7. ESWT Can Promote MSC Recruitment by Inducing Penile Tissues to Express More SDF-1 and PECAM

Finally, in order to explore other reasons why ESWT can increase MSCs, we detected SDF-1 and PECAM. Fandel et al. [23] believe that SDF-1 can attract intracavernously injected stem cells. PECAM is a chemokine like SDF-1 and can direct cell trafficking. Our results (Figures 3(b) and 3(B) and 5) indicate that after ESWT, there are lots of SDF-1 and PECAM expression in the corpus cavernosum (*P* < 0.05). So combining with the Figure 4(c), we hold the opinion that ESWT can drive recruitment of MSCs.

## 4. Discussion

DL-ESWT and MSCT have been studied as a treatment of ED for several years [45–47]. Some findings [48, 49] have been used clinically as a novel ED therapy, but translation of MSCT studies to clinic remains slow, and the molecular mechanisms of the effect of LI-ESWT remain confusing [50]. So we want to provide more theoretic and experimental bases for clinical treatment. In our study, we found that DL-ESWT could stimulate MSCs to express more VEGF in vitro and vivo. We also found that ESWT + MSCT could be a better ED therapy than by a single way. We also verified that VEGF took part in the PI3K/AKT/mTOR signaling pathway to induce autophagy and activated the NO/cGMP signaling pathway to induce vasodilatation and angiogenesis in vivo. Meanwhile, our study verified that ESWT can increase the quantity of MSCs in the corpus cavernosum.

There are many reasons for DMED, but the primary reason is vascular damage caused by oxidative stress response and hyperglycemia [35, 36]. On the one hand, vascular damage can cause penis insufficient blood supply leading to erectile disorder. On the other hand, oxidative stress response can cause neurocyte damage and endothelial apoptosis, which can reduce eNOS and nNOS in the corpus cavernosum, and these can decrease NO and then lead to DMED [51, 52]. Tepekoylu et al. [53] proved that ESWT could induce angiogenesis and lead to mobilization of endogenous endothelial cells. Prieto et al. [54] show that MSCT can stimulate angiogenesis in vascular homeostasis, and our study also shows similar consequences. What is more, our results indicate that ESWT + MSCT can improve erectile function more effectively than by a single way. Accordingly, these results confirm our hypothesis that combination of both ESWT and MSCT for ED treatment will get a better result than by a single way. In our results, we find that in vitro and vivo MSCs after ESWT can express more VEGF. So we believe that the VEGF content in the penile tissues is the key to recovering erectile function.

In some studies [23, 24], the quantity of MSCs after injection will decrease rapidly and only a few MSCs can reach the target area. They believed that most of MSCs would flow away via migration into the circulatory system. But in this experiment, we verified that ESWT could increase the quantity of MSCs in the corpus cavernosum. Also, these MSCs can be beneficial for ED treatment. Fandel et al. [23] considered that nerve injury upregulated SDF-1 expression in the ganglion and thereby attracted intracavernously injected stem cells. They injected MSCs into penile tissues and detected the labeled MSCs in the major pelvic ganglia. In our study, similarly we injected MSCs to the penile tissues, and after ESWT we found a high expression of SDF-1 and PECAM, which can drive MSCs to stay in the corpus cavernosum. We also found after ESWT the quantity of MSCs increased in the corpus cavernosum. So it is proved that ESWT could drive recruitment of MSCs. This is why ESWT can keep the MSCs in the corpus cavernosum adequately.

Some researchers [55, 56] consider that NO can trigger cavernous smooth muscle cell relaxation, which can allow blood to enter into the sinusoids during sexual arousal. They indicated that it was a crucial reason why increasing NO could improve ED. But according to the latest study, Yoshida et al. [57] consider that the level of NO in the corpus cavernosum can induce vasodilatation and stimulate angiogenesis, which is another important reason that NO could improve ED. On the basis of the NO/cGMP pathway [40, 58], we believe that more nNOS and cGMP can improve ED, efficiently. Komori et al. [59] consider that VEGF can stimulate angiogenesis by activating the NO/cGMP signaling pathway. We find that after ESWT and MSCT, VEGF expression is higher than without ESWT or MSCT. So we believe that after ESWT and MSCT the NO/cGMP signaling pathway should be activated in the corpus cavernosum. Wu et al. [60] conclude that VEGF plays an important role in NO/cGMP signaling pathway activation in the process of promoting angiogenesis. Similarly, our results demonstrated that as nNOS and cGMP in the penile tissues increase, the level of NO in the penile tissues could augment, which can relax vessels and stimulate angiogenesis, and then improve erectile function. To sum up the above arguments, we can conclude that ESWT + MSCT can be a great ED treatment because of better regeneration and repair compared with a single ESWT or MSCT.

Some researchers [22, 61] consider that there is an important relationship between apoptosis and autophagy, which means the increased autophagy can decrease apoptosis. In DMED rats' corpus cavernosum, oxidative stress response induces apoptosis [22]. The apoptosis aggravates corpus cavernosum ischemia and then weaken erectile function [62]. Autophagy [63, 64] is a self-protection that cells can get nutriment needed to sustain survival, and autophagy is an important self-regulation system of cells to inhibit apoptosis. Our results show that in the DM + ESWT + MSCT group, the number of apoptosis in the corpus cavernosum is lower than in other groups, which is close to the normal group. So we consider that ESWT + MSCT can increase autophagy to defend apoptosis in the DMED rats' corpus cavernosum. Wei et al. [37] consider that VEGF can activate the PI3K/AKT/mTOR signaling pathway and then stimulate autophagy. In our results, we found that in the DM + ESWT + MSCT group p-akt/akt expression was higher than in others. So we consider that VEGF plays an important role in the PI3K/AKT/mTOR signaling pathway in the process of improving erectile function. In brief, we believe that after being treated by DL-ESWT and MSCT, the corpus cavernosum can express a lot of VEGF, and these VEGF can bind to VEGF receptors in the corpus cavernosum. On the one hand, VEGF can stimulate the PI3K/AKT/mTOR signaling pathway, induce autophagy, and then improve erectile function. On the other hand, VEGF can trigger the NO/cGMP signaling pathway, activate vasodilatation and angiogenesis, and then improve ED. We tested the VEGF expression in each group, and the results show that the expression in the DM + ESWT + MSCT group is higher than in other groups. This result also proves that combination of LI-ESWT and MSCT can express more VEGF than a single treatment. VEGF can reach the cell surface and bind to VEGF receptors via the paracrine, which regulates autophagy and apoptosis by activating the PI3K/AKT/mTOR and NO/cGMP signaling pathway. These also explained why LI-ESWT and MSCT could induce vasodilatation and angiogenesis to improve ED. In the next experiment, we want to test the further relationship between VEGF and these two pathways. We will block the VEGF receptors on the cell surface, which cannot make VEGF and VEGF receptors combined. These experiments will find out if VEGF can trigger the PI3K/AKT/mTOR and NO/cGMP pathways exactly. Berrak et al. [65] inhibited the PI3K/AKT/mTOR pathway to test the relationship between autophagy and apoptosis in the prostate cancer cells, and some researchers [66] inhibited the NO pathway to detect the proliferation ability of endothelial cells. So next we will, respectively, inhibit the PI3K/AKT/mTOR and NO/cGMP pathways and find out these two pathways' influences on ED. If these results coincide with our results in this report, we will conclude that a combination of LI-ESWT and MSCT can be a great potential and promising way to improve ED by activating the PI3K/AKT/mTOR pathway which can increase autophagy and decrease apoptosis and the NO/cGMP pathway which can induce vasodilatation and angiogenesis.

Our experiment does have some places for improvement. Firstly, the rats in our experiment are 8 weeks old, so we do not know if LI-ESWT and MCST have the same effect on older DMED rats. Secondly, the number of MSCs in the corpus cavernosum by intracavernous injection is hard to control in each DMED rat, so it is difficult to avoid errors. At last, about the pathway, we just made an animal experiment, but no cell experiment. So in the next step, we will inhibit the PI3K/AKT/mTOR and NO/cGMP pathways and control the related gene expression to verify our animal experiment.

## 5. Conclusion

Our experiment has proved that combination of LI-ESWT and MSCT can get a better result than by a single way as a novel ED treatment. This is because combination of LI-ESWT and MSCT can express more VEGF than a single treatment, which can take part in autophagy by triggering the PI3K/AKT/mTOR signaling pathway. LI-ESWT and MSCT can induce vasodilatation and angiogenesis by activating the NO/cGMP signaling pathway. Our study has proved that ESWT can drive homing of MSCs in the corpus cavernosum. Our study provides a theoretic and experimental basis for clinical treatment. This cooperative therapy combined with stem cells and machine also provides a new research direction in ED treatment for the future.

## Data Availability

The data used to support the findings of this study are included within the article.

## Abbreviations

BCA: Bicinchoninic acid
BDNF: Brain-derived neurotrophic factor
cGMP: Cyclic guanosine monophosphate
DAPI: 4,6-Diamidino-2-phenylindole
DL-ESWT: Defocused low-energy shock wave therapy
DMED: Diabetes mellitus erectile dysfunction
ED: Erectile dysfunction
eNOS: Endothelial nitric oxide synthase
ICP: Intracavernous pressure
NGF: Nerve growth factor
NO: Nitric oxide
PDE5I: Phosphodiesterase 5 inhibitor
SDF-1: Stromal cell-derived factor-1
LC3: Light chain 3
MAP: Mean arterial blood pressure
MSCs: Mesenchymal stem cells
MSCT: Mesenchymal stem cell therapy
mTORC: Mechanistic target of rapamycin complex
nNOS: Neuronal nitric oxide synthase
PARP: Poly-ADP-ribose polymerase
PECAM: Platelet endothelial cell adhesion molecule
sGC: Soluble guanylate cyclase
STZ: Streptozotocin
VEGF: Vascular endothelial growth factor.

## Supplementary Materials

Supplementary Materials Figure 1: representative images of immunofluorescence staining after treatment of ESWT in the corpus cavernosum. Arrows were the representative area of colocalization. Original magnification: ×200. Figure 2: representative images of immunofluorescence staining after treatment of ESWT in the corpus cavernosum. Arrows were the representative area of colocalization. Original magnification: ×200.
