# Supplementary material for: Efficient Promotion of Autophagy and Angiogenesis Using Mesenchymal Stem Cell Therapy Enhanced by the Low-Energy Shock Waves in the Treatment of Erectile Dysfunction
Source: Stem Cells Int. 2018 Aug 29;2018:1302672. doi: 10.1155/2018/1302672 (PMC6136471; doi:10.1155/2018/1302672)
Supplement: Supplementary Materials — Figure 1: representative images of immunofluorescence staining after treatment of ESWT in the corpus cavernosum. Arrows were the representative area of colocalization. Original magnification: ×200. Figure 2: representative images of immunofluorescence staining after treatment of ESWT in the corpus cavernosum. Arrows were the representative area of colocalization. Original magnification: ×200. [file 1302672.f1.zip › 1302672.f1.pptx]

## Slide 1
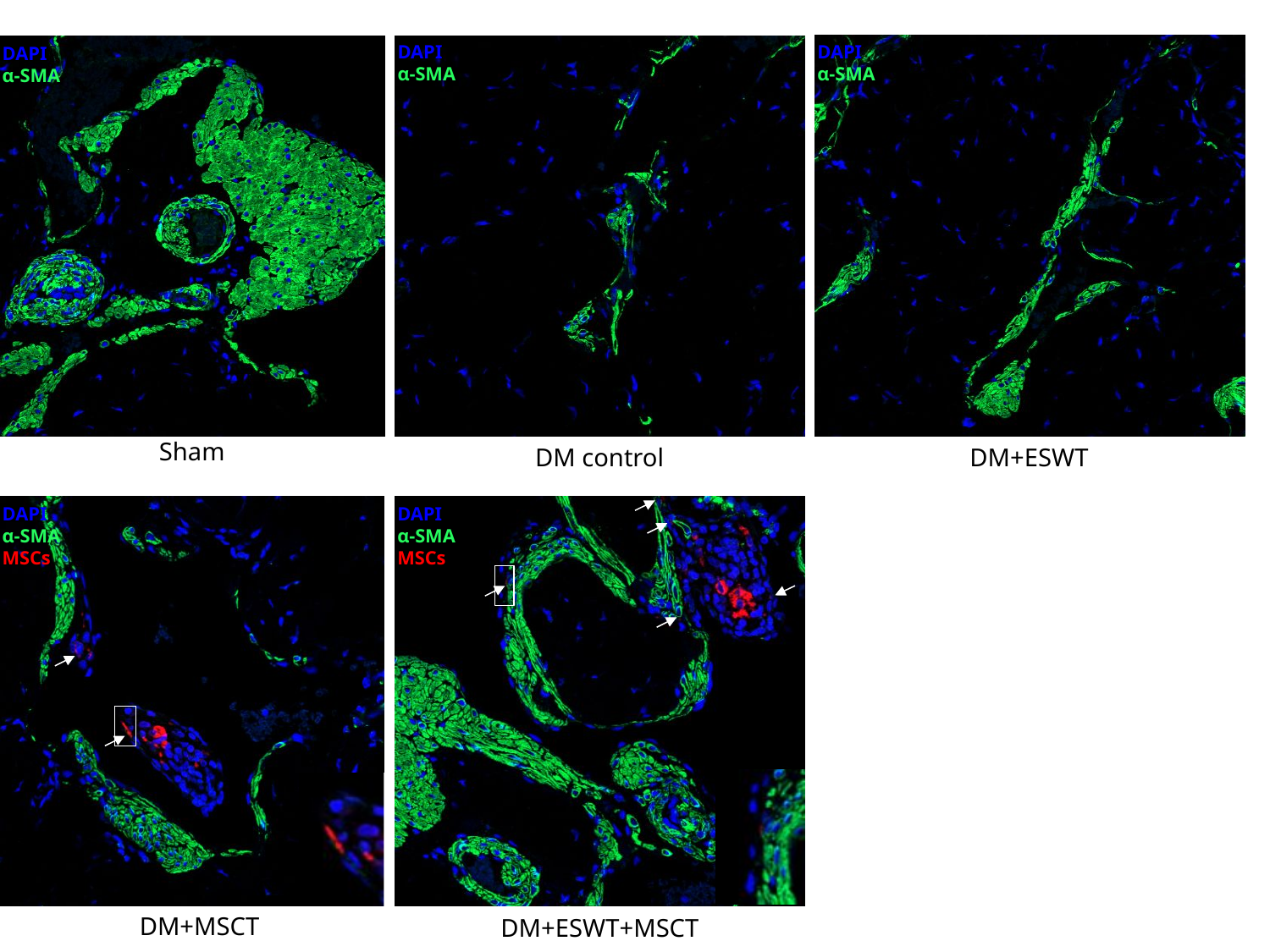

A
DAPI
α-SMA
DAPI
α-SMA
DAPI
α-SMA
Sham
DM control
DM+ESWT
DAPI
α-SMA
MSCs
DAPI
α-SMA
MSCs
DM+MSCT
DM+ESWT+MSCT
